# Consecutive Charging of a Perylene Bisimide Dye by Multistep Low‐Energy Solar‐Light‐Induced Electron Transfer Towards H_2_ Evolution

**DOI:** 10.1002/anie.202001231

**Published:** 2020-04-30

**Authors:** Yucheng Xu, Jiaxin Zheng, Joachim O. Lindner, Xinbo Wen, Nianqiang Jiang, Zhicheng Hu, Linlin Liu, Fei Huang, Frank Würthner, Zengqi Xie

**Affiliations:** ^1^ Institute of Polymer Optoelectronic Materials and Devices State Key Laboratory of Luminescent Materials and Devices South China University of Technology Guangzhou 510640 P. R. China; ^2^ Institut für Organische Chemie & Center for Nanosystems Chemistry Universität Würzburg Am Hubland 97074 Würzburg Germany

**Keywords:** electron transfer, hydrogen evolution, nanoparticles, perylene bisimides, solar energy

## Abstract

A photocatalytic system containing a perylene bisimide (PBI) dye as a photosensitizer anchored to titanium dioxide (TiO_2_) nanoparticles through carboxyl groups was constructed. Under solar‐light irradiation in the presence of sacrificial triethanolamine (TEOA) in neutral and basic conditions (pH 8.5), a reaction cascade is initiated in which the PBI molecule first absorbs green light, giving the formation of a stable radical anion (PBI^.−^), which in a second step absorbs near‐infrared light, forming a stable PBI dianion (PBI^2−^). Finally, the dianion absorbs red light and injects an electron into the TiO_2_ nanoparticle that is coated with platinum co‐catalyst for hydrogen evolution. The hydrogen evolution rates (HERs) are as high as 1216 and 1022 μmol h^−1^ g^−1^ with simulated sunlight irradiation in neutral and basic conditions, respectively.

## Graphical abstract

**The charge of the light brigade**: By a sequence of absorption and photo‐induced electron‐transfer processes electron‐poor perylene bisimides (cp‐BPI) are transformed into their electron‐rich dianions which upon another photoexcitation exhibit sufficient reductive power to drive hydrogen evolution at Pt/TiO_2_ nanoparticles.

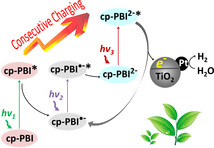

Facing the depletion of fossil fuels and increasingly severe environmental problems, clean energy technologies have drawn increasing attention in the last few decades. Splitting water into hydrogen and oxygen by photocatalysts is recognized as one of the promising strategies to utilize and store the inexhaustible solar energy.^1^, ^2^, ^3^, ^4^ Dye sensitized titanium dioxide (TiO_2_) and wide band gap metal oxides are widely used as photocatalysts because of the abundance of both organic dyes and metal oxides and the tunable energy level alignments between them, which enables the combination of efficient solar energy harvesting and charge separation capability.^5^, ^6^, ^7^, ^8^ The minimum theoretical energy threshold for water splitting is 1.23 eV, but the energy of the photons used for this process is typically larger than 2.0 eV as a result of a number of energy‐loss processes including the reorganization of the excited state of the photocatalyst system, overpotential of half reactions, and the energy lost in the charge‐transfer (redox) processes. In fact, most of the published hydrogen evolution systems only respond to blue light (*λ*<500 nm).^9^, ^10^, ^11^ There is obviously a trade‐off that wide band gap dyes can provide sufficient driving force for redox reactions but show weak light harvesting ability, while narrow band gap dyes may harvest solar energy efficiently but possess poor driving force for the redox processes.

Perylene bisimides (PBIs) are known as colorants and n‐type organic semiconductors.^12^, ^13^ Due to their outstanding light and thermal fastness PBIs have been also considered as promising organic photocatalysts recently.^14^, ^15^, ^16^, ^17^, ^18^, ^19^ Owing to the very strong electron‐withdrawing carbonyl groups, PBIs possess low frontier energy levels and can be reduced into radical anions and dianions readily by moderately strong reductive agents such as hydrazine hydrate or by very weak reductive agents with photo excitation like triethylamine.^20^, ^21^ The corresponding reduced products were shown to be stable in the absence of oxygen especially in polar solvents.^22^, ^23^, ^24^, ^25^ Interestingly, the PBI radical anions and dianions show even stronger absorption bands and cover a different absorption range compared to the neutral molecule, thereby enabling additional solar light harvesting. As a consequence, in the presence of a weak reducing agent PBI‐bound high energy electrons with a strong reducing ability may be generated by means of multiple photon absorption.^14^ Accordingly, we expect that the tunability of redox and optical properties of PBIs will enrich the currently very active field of visible light‐driven photocatalysis.^26^, ^27^, ^28^, ^29^

Herein we report the efficient usage of sunlight for hydrogen evolution through a photocatalyst system based on a perylene bisimide containing four 4‐carboxyphenoxy groups at bay positions (cp‐PBI) anchored to titanium dioxide (TiO_2_) nanoparticles loaded with platinum. With the consecutive excitation by green, infrared, and red photons, the electron‐poor neutral PBI core transformed into electron‐rich dianion by multistep photo‐induced electron transfer processes in the presence of the sacrificial TEOA agent, and then upon injection of high energy electrons into TiO_2_ nanoparticles decorated with platinum for hydrogen evolution (Figure 1). Hydrogen evolution rates (HERs) as high as 1216 and 1022 μmol h^−1^ g^−1^ were achieved with simulated sunlight irradiation in neutral and basic conditions, respectively. To our knowledge, such a consecutive charging mechanism has not yet been employed in a photocatalytic system for hydrogen generation, but has been applied by König and others for tor the photocatalytic reduction of aryl halides.^14^, ^19^, ^30^

**Figure 1:**
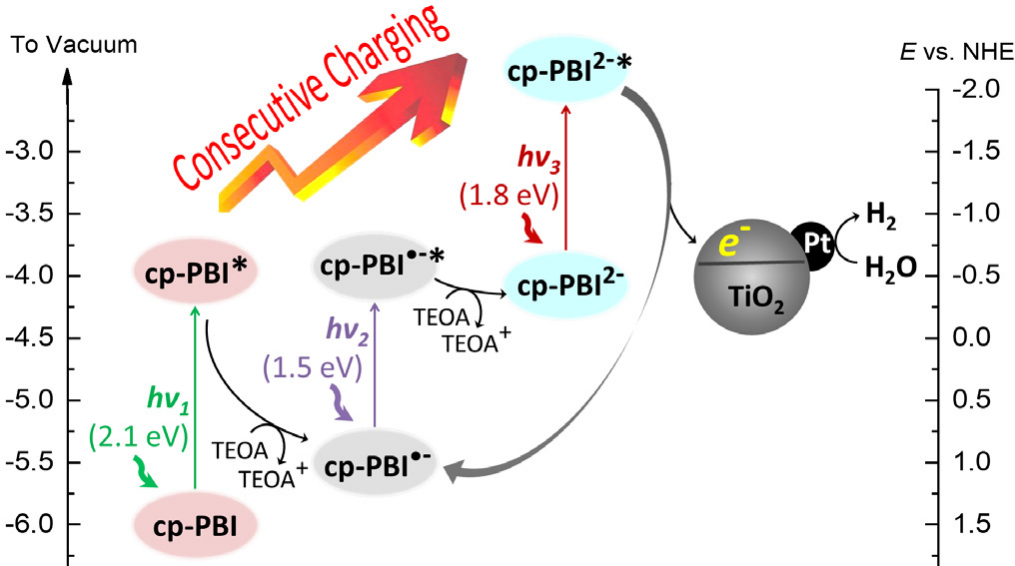
Illustration of the charging processes of cp‐PBI by multistep photo‐induced electron transfer, and the subsequent charge transfer from cp‐PBI^2−^ to TiO_2_ nanoparticle for hydrogen evolution. The positions of the energy levels for radical anionic and dianionic states are estimated based on the CV results since they cannot be directly derived from our experiments. NHE=normal hydrogen electrode.

Figure 2 a shows the chemical structure of cp‐PBI that can anchor to TiO_2_ easily through the four carboxyl groups. The frontier energy levels of cp‐PBI were analyzed by cyclic voltammetry (CV) in DMF solvent in combination with UV/Vis absorption spectroscopy (Figure S1 and S2 in the Supporting Information). cp‐PBI shows an onset reduction potential of −0.81 V versus ferrocene, which corresponds to the formation of its radical anion (cp‐PBI^.−^), while the second onset reduction potential (deduced by the first onset and the potential difference between two peaks) at −1.01 V indicates the transformation from cp‐PBI^.−^ to the dianion state (cp‐PBI^2−^). Supported by time‐dependent DFT calculations (Figure S10 and Tables S1 and S2) these two reductions can be related to the fill‐up of the cp‐PBI LUMO which now becomes the new HOMO of the cp‐PBI dianion, being more than 2.1 eV (the optical gap related to the absorption maximum of cp‐PBI) higher in energy.

**Figure 2:**
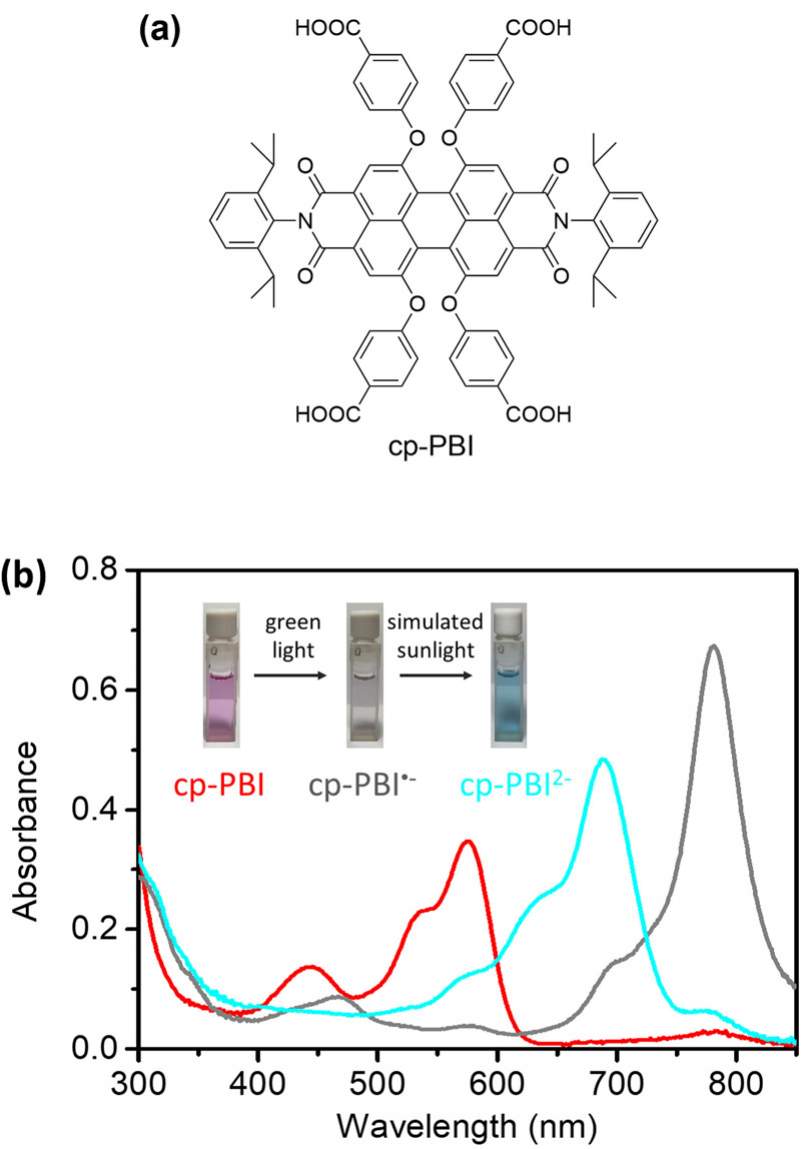
a) Chemical structure of cp‐PBI. b) UV/Vis absorption spectra of cp‐PBI (10^−5^ m) in deoxygenated DMSO in the presence of TEOA (0.1 m) in the dark, and its anion forms (cp‐PBI^.−^ and cp‐PBI^2−^) prepared by light irradiation in the presence of TEOA (0.1 m). Inset in (b) shows the photographs of the solutions in cells.

As shown in Figure 2 b, cp‐PBI shows its typical absorption spectrum with a maximum at 572 nm (red line) in the presence of TEOA in the dark, which indicates the unfavored reduction reaction due to the weak reducing ability of TEOA (*E*_(TEOA+/TEOA)_=0.52 V vs. ferrocene) in the absence of light.^3^, ^16^ However, when the reaction mixture was irradiated by a commercial green light LED (520 nm, ca. 100 mW cm^−2^, for spectrum see Figure S3), the absorption band of neutral cp‐PBI (500–600 nm) disappeared and a very strong new absorption band located around 650–850 nm emerged that is attributed to cp‐PBI^.−^ (Figure 2 b). When the above reaction mixture was further irradiated by simulated sunlight (with a 900 nm short wave pass filter), the absorption band of cp‐PBI^.−^ decreased again and a new absorption band just between those of the neutral and radical anion solutions showed up. Concomitantly the color of the solution turned into cyan, which clearly indicates the formation of cp‐PBI^2−^.^31^ The digital photographs of cp‐PBI, cp‐PBI^.−^, cp‐PBI^2−^ solutions are shown in the inset of Figure 2 b. From the absorption spectra shown in Figure 2 b and supported by our calculations (Figure S10 and Tables S1 and S2), not only a bathochromic shift but in addition an increase in absorption strength becomes obvious for cp‐PBI^2−^ compared to cp‐PBI which recommends this dianion as a promising photosensitizer.

The formations of cp‐PBI^.−^ and cp‐PBI^2−^ by photochemical method were fully verified by titration experiment using hydrazine hydrate as the reducing reagent (Figure S4). The stepwise charging processes by photochemical reaction is illustrated in Figure 1. Due to the easy reduction of cp‐PBI up to cp‐PBI^2−^ by TEOA with assistance of light irradiation and the utilization of the absorption band of cp‐PBI^2−^ at around 600–750 nm for the promotion of an electron from the highest occupied molecular orbital (HOMO) into the LUMO, a species of high reducing power is generated. Thus, this multistep photoinduced electron transfer from TEOA to cp‐PBI transforms the electron poor cp‐PBI neutral molecule gradually into an electron rich cp‐PBI^2−^ dianion, which provides high‐energy electrons after another photoexcitation. According to transient absorption spectroscopy, the life time of the excited state of the PBI radical anion is indeed quite short (145 ps) and the relaxation process into the ground state should be faster than the molecules’ diffusion process in solution.^32^, ^33^ Thus, the formation of cp‐PBI^2−^ in our experiments suggests (protonated) TEOA to be in the close vicinity of (deprotonated) cp‐PBI^.−^ (Figure S5, S6), which facilitates the charge transfer process.

To demonstrate the application of the easily generated high energy electrons bound to cp‐PBI^2−^ dianion by multiple‐step photoinduced electron transfer processes, cp‐PBI was used as a sensitizer for anatase TiO_2_ nanoparticles loaded with Pt, which is a well‐established photocatalytic system, for hydrogen evolution.^34^, ^35^ The preparation of Pt/TiO_2_/cp‐PBI nanoparticles is described in the Supporting Information. The diameter of the Pt/TiO_2_/cp‐PBI clusters was determined to be about 30 nm by SEM and dynamic light scattering (DLS), which is much larger than the pristine TiO_2_ nanoparticles (Figure S7). Figure S8 shows the diffuse reflection spectra (DRS) pattern of TiO_2_, Pt/TiO_2_ and Pt/TiO_2_/cp‐PBI complex, respectively. TiO_2_ does not show any absorption over the whole visible light region and after loading with Pt the absorption is only slightly red‐shifted. However, the absorption of Pt/TiO_2_/cp‐PBI was widely extended into the visible light region which is attributed to the absorption of cp‐PBI. Compared with the absorption spectrum of cp‐PBI in DMSO solution, each absorption peak of Pt/TiO_2_/cp‐PBI in the visible light region remained in their original position indicating the absence of aggregation by intermolecular π–π stacking.^36^

Photocatalytic experiments were then carried out in water (pH 7.0 or 8.5) under simulated sunlight irradiation with TEOA as sacrificial electron donor. The results are shown in Figure 3 and Table 1. The HERs of Pt/TiO_2_/cp‐PBI reached 1216 μmol h^−1^ g^−1^ and 1022 μmol h^−1^ g^−1^ in neutral and basic condition with turnover frequencies (TOFs) of 245 h^−1^ and 206 h^−1^, respectively. A control experiment using Pt/TiO_2_ nanoparticles as photocatalyst was also conducted, and the HER was observed to be only about 1/10 to that of Pt/TiO_2_/cp‐PBI, implying the vital role of the cp‐PBI on the HER. This performance is comparable to the best results reported for conventional photosensitizer dyes including far more expensive ruthenium bipyridine based systems.^37^ The durability of Pt/TiO_2_/cp‐PBI was further tested in the recycled (degassed every 5 hours) hydrogen evolution experiment, and the result showed that the HER remained 80 % of the initial value after 20 hours of irradiation (Figure 3 b). During the reaction process the color of the system is cyan (Figure 3 c), while after exposure to air it changed back to the initial red color. These results demonstrate that the involved photo‐reduced PBI species are like their neutral precursors endowed with a high stability.

**Figure 3:**
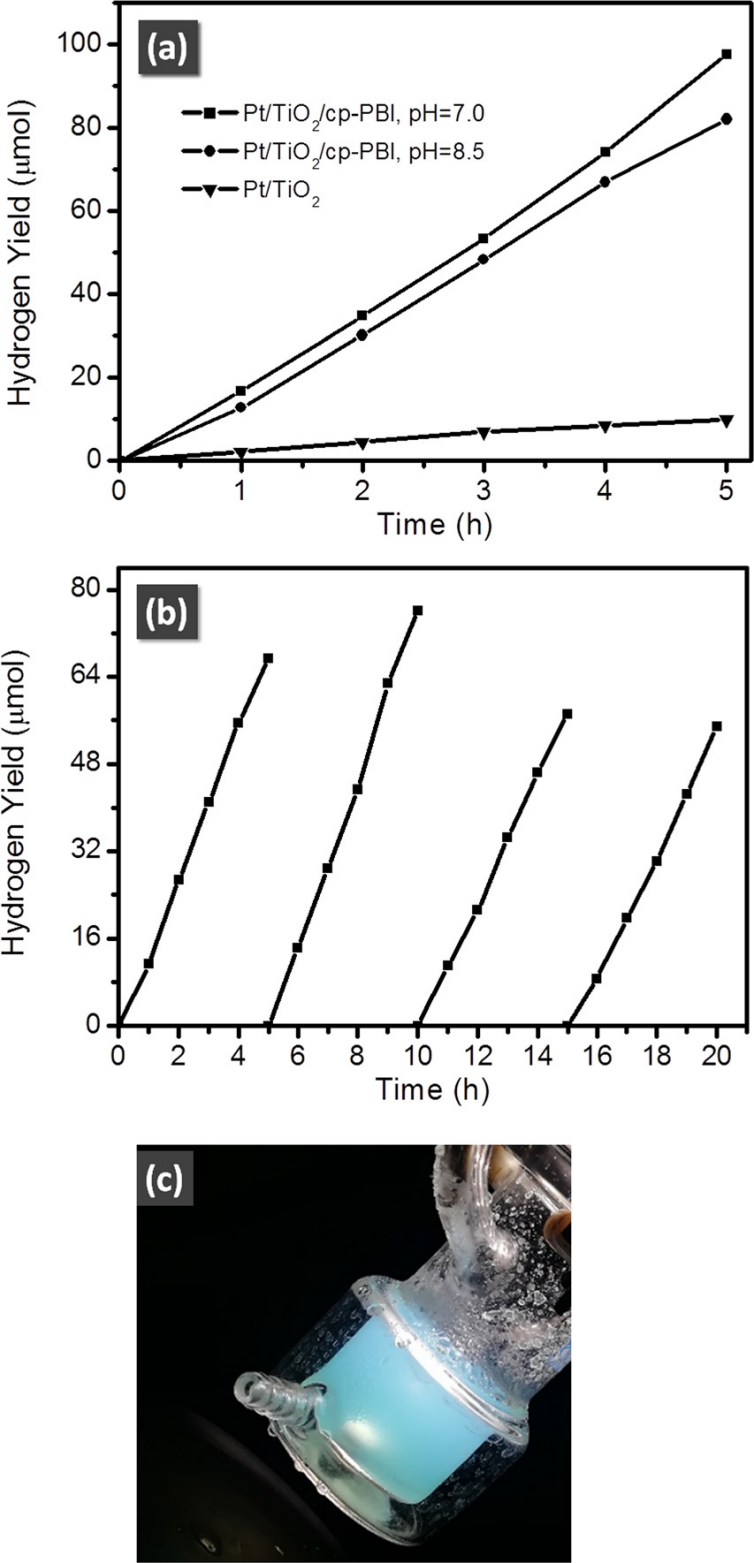
a) Photocatalytic activity of Pt/TiO_2_/cp‐PBI for hydrogen evolution in 5 hours. Conditions: 20 mg of sample in 50 mL of TEOA (0.1 m) aqueous solution. A 300 W Xe lamp (AM 1.5 filter, 100 mW cm^−1−2^) was used as a light source. b) Long term experiment of Pt/TiO_2_/cp‐PBI using TEOA (0.1 m) as sacrificial agent (pH 7.0). Samples were degassed every five hours. c) Photograph of the reaction mixture of Pt/TiO_2_/cp‐PBI during hydrogen evolution experiment.

**Table 1:** Photocatalytic performance of Pt/TiO_2_/cp‐PBI under various conditions with TEOA as a sacrificial agent.

Reaction conditions			HER [μmol h^−1^ g^−1^]	TOF [h^−1^]^[a]^
Light source	pH	Reaction time [h]		
AM 1.5	7.0	5	1216	245
AM 1.5	8.5	5	1022	206
AM 1.5 with light filter (*λ*<600 nm)	7.0	3	24	3.1
AM 1.5 with light filter (*λ*>600 nm)	7.0	3	2.5	0.6

[a] TOF is calculated with respect to the amount of dye.

Because the color of the reaction mixture became cyan during the photocatalytic experiment (Figure 3 c), we hypothesized that the active species involved in the hydrogen generation process is not the cp‐PBI radical anion (which has already a very high reducing power) but the cp‐PBI dianion (with its even higher reducing power, see Figure 1). To confirm this hypothesis, we recorded the DRS spectra of a reaction mixture that was deoxygenated carefully, in which the concentration of Pt/TiO_2_/cp‐PBI was 500 times higher than that used in typical experiments to give an evident DRS signal. The DRS spectrum of Pt/TiO_2_/cp‐PBI with TEOA in dark was similar to that of powders as shown in Figure S9, showing an absorption maximum at 583 nm that was attributed to cp‐PBI. After the mixture was irradiated by simulated sunlight for 1 hour, the absorption of cp‐PBI decreased and two new absorption bands with peaks at 780 nm and 658 nm emerged. The band at 780 nm was ascribed to cp‐PBI^.−^, and the band at 658 nm was ascribed to cp‐PBI^2−^, despite relatively large blue shifts when compared with those in DMSO solution. These results implied that both cp‐PBI^.−^ and cp‐PBI^2−^ were generated during the photocatalytic processes. Furthermore, two control experiments were performed for Pt/TiO_2_/cp‐PBI, one with 600 nm short wave pass filter to avoid the excitation of the cp‐PBI^.−^ and the other one with a 600 nm long wave pass filter to avoid the excitation of the cp‐PBI. The results indicated that only very slow HERs were observed in both cases (Table 1), which confirms our hypothesis that the Pt/TiO_2_/cp‐PBI photocatalyst system operates in the presence of TEOA under both neutral and basic conditions via a consecutive electron‐transfer process as illustrated in Figure 1.

In summary, a perylene bisimide equipped with four 4‐carboxyphenoxy groups at bay positions exhibits a suitable combination of coordination and functional properties for its application as a photosensitizer for solar‐light‐induced hydrogen evolution. In the polar solvents DMSO and water a multi‐step photo‐induced electron transfer from sacrificial electron‐donor molecules to cp‐PBI molecules was observed, leading to the generation of PBI radical anions and dianions. These could be successfully applied in the photocatalytic system of Pt/TiO_2_/cp‐PBI under neutral and basic conditions. Thus, TiO_2_‐bound dye molecules absorbed low energy photons of different colors (green, near‐infrared, and red) gradually to generate sufficiently high‐energy electrons for the charging of Pt/TiO_2_ for water reduction. The hydrogen evolution rates as high as 1216 and 1022 μmol h^−1^ g^−1^ were achieved under neutral and basic conditions, respectively. These results demonstrate a new strategy for photocatalytic systems based on in situ generated strongly absorbing dianions from electron‐deficient color pigments.^38^ Further research on the sacrificial agent free light‐driven systems for photocatalytic water splitting^39^ is ongoing.

## Conflict of interest

The authors declare no conflict of interest.

## Supporting information

As a service to our authors and readers, this journal provides supporting information supplied by the authors. Such materials are peer reviewed and may be re‐organized for online delivery, but are not copy‐edited or typeset. Technical support issues arising from supporting information (other than missing files) should be addressed to the authors.

Supplementary
