# Supplementary material for: Consecutive Charging of a Perylene Bisimide Dye by Multistep Low‐Energy Solar‐Light‐Induced Electron Transfer Towards H2 Evolution
Source: Angew Chem Int Ed Engl. 2020 Apr 30;59(26):10363–7. doi: 10.1002/anie.202001231 (PMC7317913; doi:10.1002/anie.202001231)
Supplement: Supplementary file 1 — Supplementary [file ANIE-59-10363-s001.pdf]

Angewandte Chemie
Eine Zeitschrift der Gesellschaft Deutscher Chemiker

Supporting Information

Consecutive Charging of a Perylene Bisimide Dye by Multistep Low-Energy Solar-Light-Induced Electron Transfer Towards H_2 Evolution

Yucheng Xu, Jiaxin Zheng, Joachim O. Lindner, Xinbo Wen, Nianqiang Jiang, Zhicheng Hu, Linlin Liu, Fei Huang, Frank Würthner, and Zengqi Xie**

anie_202001231_sm_miscellaneous_information.pdf

Table of Contents

1. Materials and Synthesis S3
2. Characterization S3
3. Photocatalytic H_2 Evolution Experiments S3
4. Cyclic Voltammetry Curve of cp-PBI S4
5. UV-vis and Photoluminescence Spectroscopy of cp-PBI S4
6. Luminescence Spectra of the LED for cp-PBI$^{\bullet -}$ Preparation S4
7. UV-vis Spectra During Reduction Process of cp-PBI with Hydrazine Hydrate as Reducing Reagent S5
8. Comparison of cp-PBI and mp-PBI S5
9. Basic Information of Pt/TiO_2/cp-PBI Nanoparticles: XRD, DLS and SEM S7
10. DRS Spectrum of TiO_2, Pt/TiO_2 and Pt/TiO_2/cp-PBI Complex S8
11. DRS Spectrum of Pt/TiO_2/cp-PBI During Hydrogen Evolution Experiment S8
12. DFT Calculations S8
References S9

1. Materials and Synthesis

Materials

All materials and reagents used in this work were purchased from Sigma-Aldrich and used as received without further purification.

Synthesis of PBI, cp-PBI$^{\bullet-}$ and cp-PBI^{2-}

cp-PBI was synthesized following reported route.[S1] cp-PBI$^{\bullet-}$ can be prepared by dissolving 100 μL of TEOA and 10^{-5} M of cp-PBI in 3 mL of DMSO. The reaction mixture was deoxygenated and irradiated by a commercial green light LED (500 nm, ≈100 mW/cm^2), resulting in the formation of cp-PBI$^{\bullet-}$. cp-PBI^{2-} can be obtained when the above mixture was irradiated by simulated sunlight.

In the chemical route, 10^{-5} M of cp-PBI was dissolved in 3 mL of DMSO in the presence of hydrazine hydrate (670 μL). Hydrazine hydrate first reacted to give a colourless solution of cp-PBI anions and to reduce air. Increasing the amount of hydrazine hydrate to 1500 μL give a blue solution (Fig. S8a), indicating the formation of cp-PBI^{2-}.

Synthesis of TiO_2 Nanoparticles

TiO_2 nanoparticles were synthesized following the previously reported method.[S2] Typically, 8 mL of anhydrous ethanol was cooled down to 0 ℃, and 2 mL of $TiCl_4$ was added with virous stirring. The above solution was then transferred into 40 mL of benzyl alcohol. The solution was stirred at 80 ℃ for 9 hours. After the reaction was completed, the reaction mixture was diluted by addition of 100 mL of diethyl ether and white TiO_2 powders precipitated immediately. The TiO_2 powder was collected by centrifuge, washed by diethyl ether 3 times, and redispersed in ethanol as stock solution with a concentration of 5 mg/mL.

Synthesis of Pt/TiO_2/cp-PBI

cp-PBI is insoluble in neutral water, but is well soluble in ethanol. Upon addition of cp-PBI in ethanol into the stock solution of TiO_2 nanoparticles in ethanol, red-colored precipitates form within several minutes, indicating cp-PBI anchoring on the TiO_2 nanoparticle surface via carboxyl groups. In order to synthesize Pt/TiO_2/cp-PBI, Pt was accordingly firstly loaded onto the TiO_2 nanoparticles according to a photodeposition pathway as described elsewhere.[S3] Briefly, 2.64 mg of $H_2PtCl_6 \cdot 6H_2O$ was added into 20 mL of above stock solution. The reaction mixture was stirred and irradiated with a 500 W Hg lamp in Ar atmosphere for 1 hour. The colourless solution changed into dark grey, indicating that Pt was successfully loaded onto TiO_2 nanoparticles. After the reaction finished, 1 mg of cp-PBI was added into the reaction mixture, and the solution began to precipitate within several minutes. The reaction mixture was kept overnight to enable full adsorption of cp-PBI. The precipitate was then collected by centrifuge, washed with ethanol 2 times, and redispersed in deionized water as stock solution with a concentration of 5 mg/mL.

2. Characterization

X-ray diffraction (XRD) pattern was obtained from powder TiO_2 (the stock solution was dried in vacuum and room temperature) using a X'Pert Pro MPD X-ray diffractometer. Dynamic light scattering (DLS) and zeta potential measurement were carried out with a Zetasizer Nano ZSE analyser. UV-vis absorption and diffuse reflectance absorption spectra were obtained using a SHIMADZU UV-3600 UV-VIS-NIR spectrophotometer. Photoluminescence spectra were obtained with a SHIMADZU RF-5301PC spectrophotometer. Energy level of cp-PBI was analysed by cyclic voltammetry on a CHI760E electrochemical analyser using Pt plate, glassy carbon electrode and Ag^+/Ag as counter, working and reference electrode, respectively. The experiment was carried out in N_2 atmosphere with Bu_4NPF_6 solution in DMF as electrolyte. Ferrocene/ferrocenium (Fc/Fc^+), whose redox potential was assigned to -4.8 V vs vacuum level, was used as an internal standard. The scanning rate was 100 mV/min.

3. Photocatalytic H_2 Evolution Experiments

The photocatalytic hydrogen evolution experiments were carried out at room temperature on a Labsolar-IIIAG photocatalytic system (Perlight, Beijing) with 50 mL reactor. Typically, 4mL of Pt/TiO_2/cp-PBI stock dispersion was added into 45 mL of deionized water. 0.1M of TEOA was dissolved in reaction mixture. The pH of the reaction solution was adjusted to 7.0 or 8.5 with HCl. The reaction mixture was degassed before experiment. A 300 W Xe lamp (Ceaulight) with AM 1.5 filter was used as the light source. The optical power density at the surface of the reaction solution was adjusted to 100 mW/cm^2 by a power meter. Hydrogen was detected using gas chromatography (GC7900II, TCD detector, 5 Å molecular sieve columns, using Ar as carrier gas, standardized by injecting different volumes of hydrogen gas before each experiment).

4. Cyclic Voltammetry Curve of cp-PBI

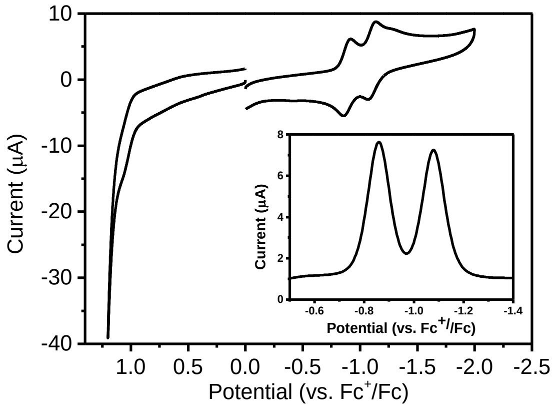

Figure S1. Cyclic voltammetry and differential pulse voltammogram (inset) curves of cp-PBI in DMF with Bu_4NPF_6 as electrolyte. Conditions: cp-PBI ca. 1mg/mL, Bu_4NPF_6 38.7mg/mL, scan rate 100 mV/min.

5. UV-vis and Photoluminescence Spectroscopy of cp-PBI

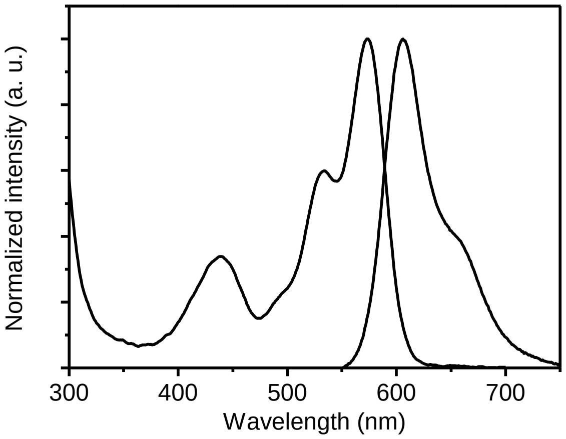

Figure S2. UV-vis absorption spectrum and photoluminescence spectrum of cp-PBI in DMSO. Conditions: cp-PBI 10^{-5} M, excited at 500 nm in PL spectrum.

6. Luminescence Spectra of the LED for cp-PBI$^{\bullet-}$ Preparation

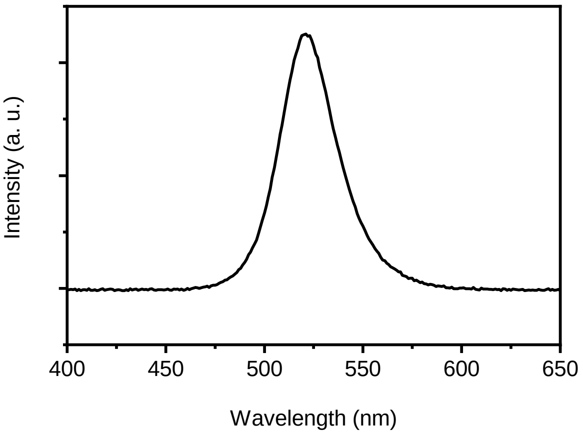

Figure S3. Spectrum of the green light LED, which was used to prepare cp-PBI$^{\bullet-}$.

7. UV-vis Spectra During Reduction Process of cp-PBI with Hydrazine Hydrate as Reducing Reagent

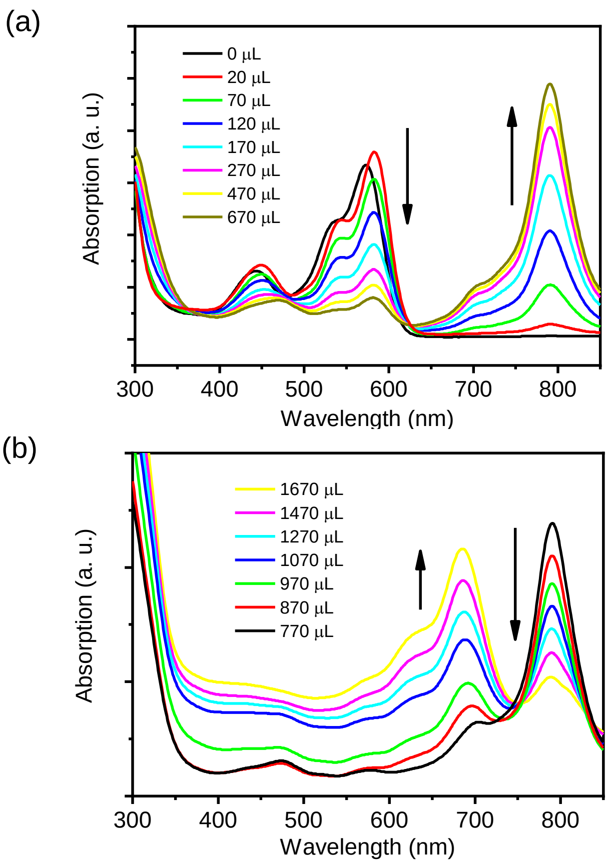

Figure S4. UV-vis absorption spectra when adding hydrazine hydrate into cp-PBI solution in DMSO. The first process of cp-PBI$^{\bullet-}$ formation is shown in a) and the second process of cp-PBI^{2-} formation in b). Conditions: cp-PBI 10^{-5} M, DMSO 3mL. The legend in picture represents how much hydrazine was added.

8. Comparison of cp-PBI and mp-PBI

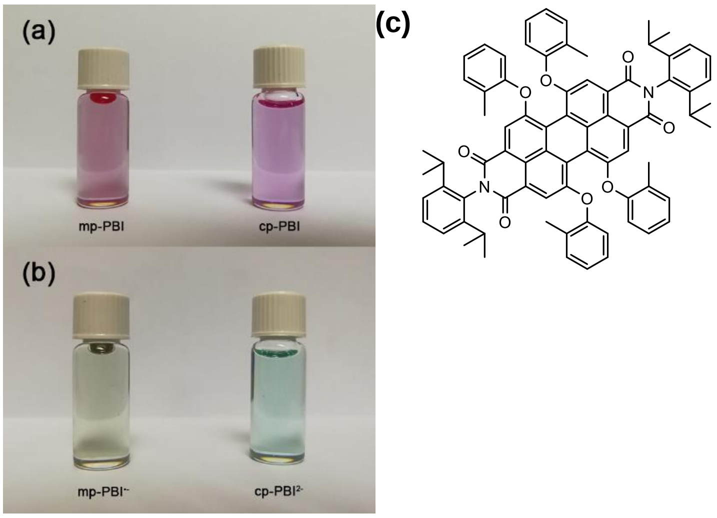

Figure S5. Digital photos of cp-PBI and mp-PBI solutions (a) before and (b) after irradiation, conditions: 10^{-5} M in DMSO, 0.1 M of TEOA, deoxygenated, AM 1.5, 100mW/cm^{-2}, 2 min. Bearing four carboxy groups, cp-PBI was easily charged to cp-PBI^{2-}, while the formation of mp-PBI^{2-} was slower according to the change of the colors of the solution. (c) Chemical structure of mp-PBI.

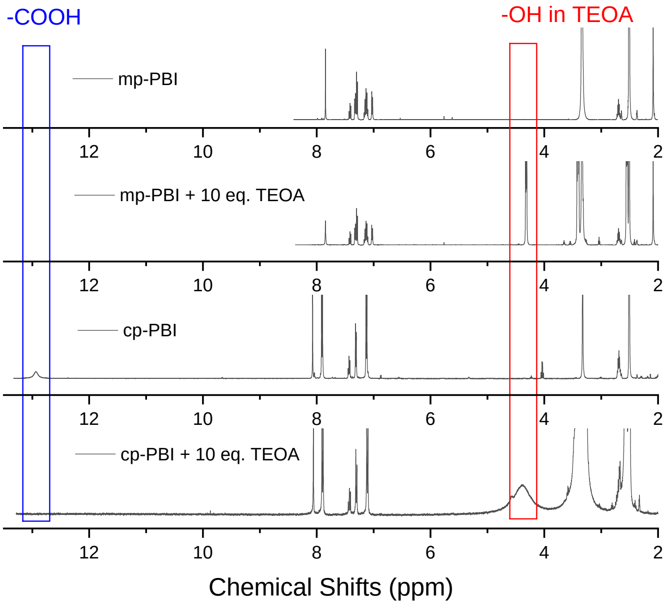

Figure S6. ^{1}H-NMR spectra of mp-PBI (0.5 mg in 0.5 mL d-DMSO), mp-PBI + 10 eq. TEOA (0.5 mg mp-PBI and 0.582 μL TEOA in 0.5 mL d-DMSO), cp-PBI (0.5 mg in 0.5 mL d-DMSO) and cp-PBI + 10 eq. TEOA (0.5 mg cp-PBI and 0.529 μL TEOA in 0.5 mL d-DMSO). The protons of TEOH hydroxy groups show a sharp signal for the sample of mp-PBI + 10 eq. TEOA. In contrast, the protons of TEOH hydroxy groups show a broad signal for the sample of cp-PBI + 10 eq. TEOA, indicating proton exchange between the two species. In addition, the COOH signal located at 12.9 ppm disappears for the sample of cp-PBI + 10 eq. TEOA, indicating that the carboxyl groups were ionized to give carboxylate / protonated TEOA complexes.

9. Basic Information of Pt/TiO_2/cp-PBI Nanoparticles: XRD, DLS and SEM

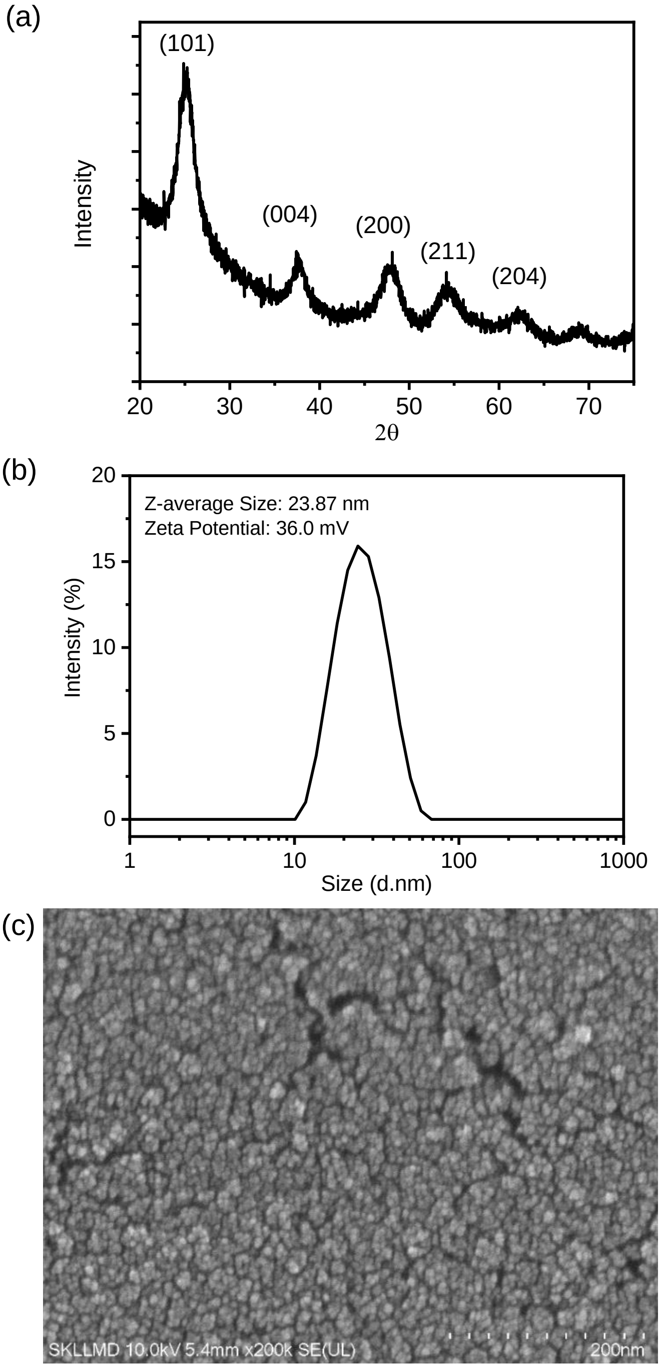

Figure S7. (a) X-ray diffraction pattern of TiO_2 nanoparticles. The diameter of TiO2 particles was determined to be 4.8 nm by Debye-Scherrer equation. (b) Dynamic light scattering spectra and (c) SEM picture of Pt/TiO_2/cp-PBI. Conditions: XRD and SEM were performed directly with powders. DLS was carried out with aqueous solution (5mg/mL).

10. DRS Spectrum of TiO_2, Pt/TiO_2 and Pt/TiO_2/cp-PBI Complex

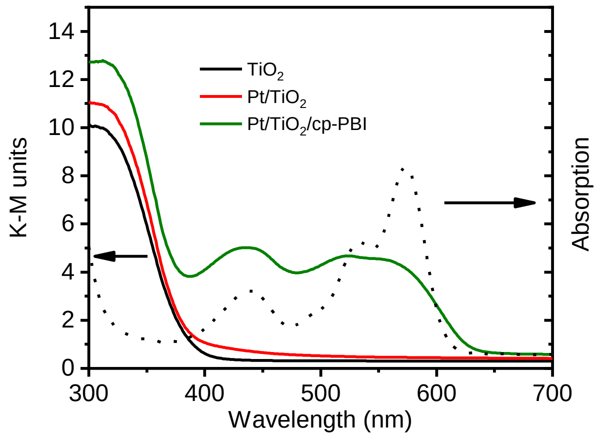

Figure S8. DRS spectrum of TiO_2, Pt/TiO_2 and Pt/TiO_2/cp-PBI complex (powder on quartz substrate). UV-vis spectrum of cp-PBI in DMSO (10^{-5} M) (dash line) was also included for comparison.

11. DRS Spectrum of Pt/TiO_2/cp-PBI During Hydrogen Evolution Experiment

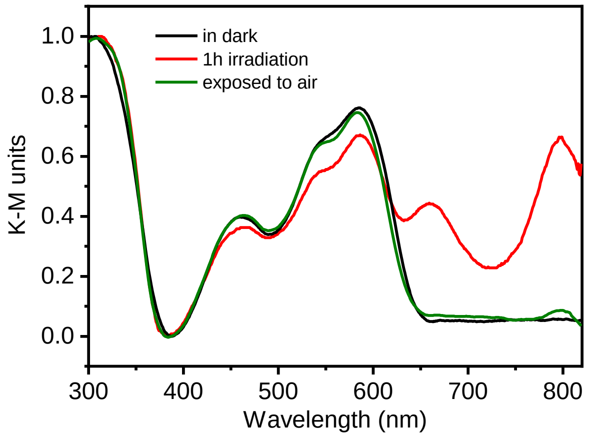

Figure S9. DRS spectrum of Pt/TiO_2/cp-PBI (0.2 g/mL) in deoxygenated water in the presence of TEOA (0.1M, pH = 7.0). For the evidence of signal concentration of Pt/TiO_2/cp-PBI was 500 times higher than that used in typical experiments.

12. DFT Calculations

For the DFT studies, a tetraphenoxy perylene bisimide model compound (with methyl groups at imide position and no carboxylic acid ligands at the phenoxy substituents), see Fig. S9. Geometries have been optimized for the neutral and dianionic systems using the B3LYP functional[S4,S5] together with the def2-SVP basis set[S6] as implemented in Gaussian 16.[S7] After verification as stationary points by vibrational frequency analysis, these optimized structures have been used as a basis for single point TD-DFT calculations using the long-range corrected CAM-B3LYP functional[S8] with the same basis set.

Table S1. Transition energy E, wavelength λ and oscillator strength f of the lowest three electronic excitations as calculated for the neutral cp-PBI model.

Transition	E / eV	λ / nm	f
**1**	2.48	499	0.62
**2**	3.37	368	0.32
**3**	3.42	362	0.03

Table S2. Transition energy E, wavelength λ and oscillator strength f of the lowest three electronic excitations as calculated for the dianionic cp-PBI^{2-} model.

Transition	E / eV	λ / nm	f
**1**	2.23	556	0.75
**2**	2.26	548	0.19
**3**	2.76	450	0.04

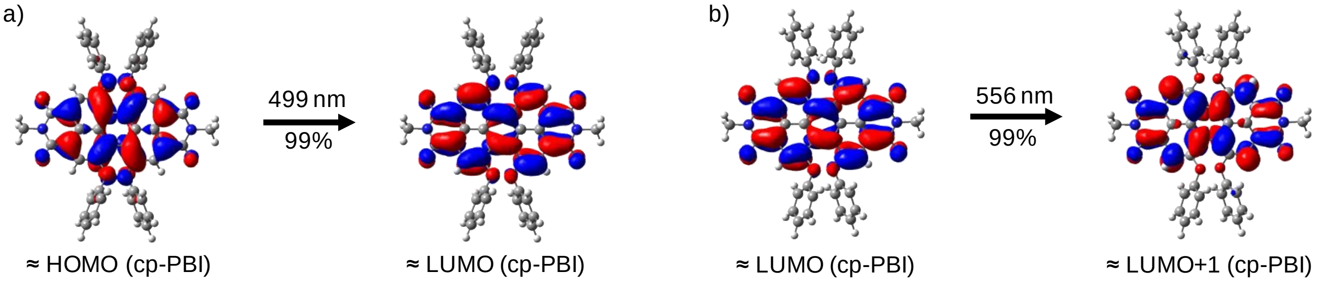

Figure S10. Natural transition orbitals of the lowest electronic transitions in a) the cp-PBI model and b) the cp-PBI^{2-} model. The percentage of the depicted excitation in the total character of the transition is given below the arrows. Additionally, a comparison to the frontier orbitals of the neutral cp-PBI model is provided below the orbital pictures.

References

[S1] S. Yang, X. Shi, S. Park, S. Doganay, T. Ha, S. C. Zimmerman, *J. Am. Chem. Soc.* **2011**, *133*, 9964-9967.

[S2] K. Wojciechowski, M. Saliba, T. Leijtens, A. Abate, H. J. Snaith, *Energ. Environ. Sci.* **2014**, *7*, 1142-1147.

[S3] A. Tiwari, N. Duvva, V. N. Rao, S. M. Venkatakrishnan, L. Giribabu, U. Pal, *J. Phys. Chem. C* **2019**, *123*, 70-81.

[S4] A. D. Becke, *J. Chem. Phys.* **1993**, *98*, 5648-5652.

[S5] C. Lee, W. Yang, R. G. Parr, *Phys. Rev. B* **1988**, *37*, 785-789.

[S6] F. Weigend, R. Ahlrichs, *Phys. Chem. Chem. Phys.* **2005**, *7*, 3297-3305.

[S7] Gaussian 16, Revision A.03, M. J. Frisch, G. W. Trucks, H. B. Schlegel, G. E. Scuseria, M. A. Robb, J. R. Cheeseman, G. Scalmani, V. Barone, G. A. Petersson, H. Nakatsuji, X. Li, M. Caricato, A. V. Marenich, J. Bloino, B. G. Janesko, R. Gomperts, B. Mennucci, H. P. Hratchian, J. V. Ortiz, A. F. Izmaylov, J. L. Sonnenberg, D. Williams-Young, F. Ding, F. Lipparini, F. Egidi, J. Goings, B. Peng, A. Petrone, T. Henderson, D. Ranasinghe, V. G. Zakrzewski, J. Gao, N. Rega, G. Zheng, W. Liang, M. Hada, M. Ehara, K. Toyota, R. Fukuda, J. Hasegawa, M. Ishida, T. Nakajima, Y. Honda, O. Kitao, H. Nakai, T. Vreven, K. Throssell, J. A. Montgomery, Jr., J. E. Peralta, F. Ogliaro, M. J. Bearpark, J. J. Heyd, E. N. Brothers, K. N. Kudin, V. N. Staroverov, T. A. Keith, R. Kobayashi, J. Normand, K. Raghavachari, A. P. Rendell, J. C. Burant, S. S. Iyengar, J. Tomasi, M. Cossi, J. M. Millam, M. Klene, C. Adamo, R. Cammi, J. W. Ochterski, R. L. Martin, K. Morokuma, O. Farkas, J. B. Foresman, D. J. Fox, Gaussian, Inc., Wallingford CT, **2016**.

[S8] T. Yanai, D. Tew, N. Handy, *Chem. Phys. Lett.* **2004**, *393*, 51-57.
